# Investigation on the effect of noisy galvanic vestibular stimulation on fine motor skills during a visuomotor task in healthy participants

**DOI:** 10.1371/journal.pone.0216214

**Published:** 2019-05-02

**Authors:** Eunice Kuatsjah, Mahta Khoshnam, Carlo Menon

**Affiliations:** Menrva Research Group, Schools of Mechatronic Systems Engineering and Engineering Science, Simon Fraser University, Metro Vancouver, British Columbia, Canada; University of Ontario Institute of Technology, CANADA

## Abstract

Noisy galvanic vestibular stimulation (nGVS) has been shown to improve dynamic walking stability, affect postural responses, enhance balance in healthy subjects, and influence motor performance in individuals with Parkinson’s disease. Although the studies to fully characterize the effect of nGVS are still ongoing, stochastic resonance theory which states that the addition of noisy signal may enhance a weak sensory input signals transmission in a non-linear system may provide a possible explanation for the observed positive effects of nGVS. This study explores the effect of nGVS on fine tracking behavior in healthy subjects. Ten healthy participants performed a computer-based visuomotor task by controlling an object with a joystick to follow an amplitude-modulated signal path while simultaneously receiving a sham or pink noise nGVS. The stimulation was generated to have a zero-mean, linearly detrended 1/f-type power spectrum, Gaussian distribution within 0.1–10 Hz range, and a standard deviation (SD) set to 90% based on each participant’s cutaneous threshold value. Results show that simultaneous nGVS delivery statistically improved the tracking performance with a decreased root-mean-squared error of 5.71±6.20% (mean±SD), a decreased time delay of 11.88±9.66% (mean±SD), and an increased signal-to-noise ratio of 2.93% (median, interquartile range (IQR) 3.31%). This study showed evidence that nGVS may be beneficial in improving sensorimotor performance during a fine motor tracking task requiring fine wrist movement in healthy subjects. Further research with a more comprehensive subset of tasks is required to fully characterize the effects of nGVS on fine motor skills.

## Introduction

Visuomotor skill plays an important role in performing daily activities. This skill requires combining sensory information across modalities and transforming them into the appropriate motor response [1]. The visuomotor process manifests as different activities ranging from executing basic functions such as walking, navigating, and grasping, to more complex activities such as driving a car, playing sports, and operating machinery and robots. Moreover, in the occupational field, robotic teleoperation has been increasingly used to perform tasks that require precision as well as to overcome geographical barriers such as in telesurgery, or in situations where the tasks pose dangers to humans if they are physically present in the field [2]. Some examples of the latter include bomb disposal/mine clearing robots [3], repair robots in space [4], and nuclear material and hazardous waste handling robots [5,6]. Many of these teleoperated robotic applications may require precise control of fine motor movements from the human operator to effectively accomplish a task.

Although generally known to be responsible for balance function, the vestibular system is strongly multimodal and highly convergent with other sensory and motor signals contributing to a diverse range of functions such as automatic reflexes, spatial coordination, and motor planning [7], spatial navigation, learning, and memory [8–10], spatial cognition, body representation, affective processes [11], bodily self-consciousness [12], and self-motion perception [13]. The vestibular end organs communicate with the brainstem and the cerebellum through the vestibular branch of the eight cranial nerve that projects to the vestibular nuclei which integrate input from the cerebellum, visual, somatic sensory systems [14]. The vestibular nuclei projects to several brain areas including the cerebellum, thalamus, and cortex and carries information regarding movement and body position that influence motor function [14]. For example, the vestibulocerebellum receives input from the vestibular nuclei for primarily maintaining equilibrium, oculomotor control, and muscle tone [14,15]. Dysfunction of the flocculus of the vestibulocerebellum typically results in gaze control abnormalities such as poor visual tracking of a moving object, as well as periodic attacks that affect fine motor abilities [16,17]. Vestibular signals are pervasive throughout the central nervous system and cortex [14]. However, parieto-insular vestibular cortex (PIVC) region has been shown to receive prominent vestibular input where many of its neuron activities correlate to proprioception [18] and visual tracking of a moving target [19]. This region has been shown to contain multisensory convergence information of self-motion cues with external visual object motion and that it may define the relative movement of the head, body, and external visual objects in space [20].

The relation between vestibular information and motor output has also been previously studied based on the framework of sensorimotor transformation required for a reflex generation [7,21,22] which is integral for producing accurate motion of the human body. For example, vestibulo-ocular reflex is driven by vestibular signals from the inner ear to produce eye movements for stabilizing images on the retina to compensate for the slightest head movements that are present during everyday activities [14]. This reflex is crucial for proper function of gaze control which relies on visual, vestibular, and proprioceptive interactions that occur throughout the central vestibular pathways [7]. This combined sensory information along with compensatory motor commands are also required for adjusting movements should a deviation occurs from the desired path [23]. Sensory feedback from the visual, vestibular, and somatosensory systems provide information about the body’s position in space, and this sensory information is further passed on to the premotor descending neurons that connect to various motor centers to produce the appropriate motor action [23]. Although the exact mechanism of vestibular processes in different brain areas related to self-motion and spatial orientation is yet to be fully understood, the projected vestibular signals would be expected to play an important role in fine visuomotor control.

Galvanic Vestibular Stimulation (GVS) is a non-invasive technique which involves sending electrical stimulation to the vestibular nerve through an electrode placed on the mastoid area. GVS bypasses the hair cells and alters the firing rate of vestibular afferents that innervate underneath the mastoids towards the brainstem nuclei and has been consistently shown to activate subcortical and cortical regions related to the vestibular system [24–31]. Previous fMRI studies have shown that GVS is able to elicit activations in the precentral and post central gyri, supplementary motor area, inferior parietal lobule, the insula, central sulcus, middle and inferior frontal gyri, superior temporal gyrus, cingulate cortex, and in the cerebellum [28,32]. In another fMRI study, a significant interaction of GVS with the neural mechanisms underlying allocentric visuospatial judgments was also observed in the right posterior parietal and ventral premotor cortex [33].

In healthy subjects, noisy GVS (nGVS) has shown to improve the dynamic walking stability [34], affect postural responses [35], improve locomotor stability [36], and enhance balance [37,38]. In individuals with Parkinson’s disease (PD), nGVS has shown to improve autonomic and motor responsiveness [39], influence motor performance on manual tracking behavior [40], and improve balance and motor symptoms. A previous study reported that nGVS is able to modulate neural oscillations in healthy subjects, and this proposed mechanism is thought to be a potential explanation for the previously observed cognitive and motor effects of vestibular stimulation [41] such as enhanced visual memory [42] and motor function [39]. The observed modulated EEG activity due to nGVS may be explained by the modulation of vestibular processing areas as well as the indirect effect on the cortical and subcortical activity due to the trans-synaptic propagation from the target region of vestibular end organs [41,43]. The complex thalamocortical loops are thought to be manipulated from receiving the input from the vestibular afferent projections influenced by nGVS [41,44,45]. Past studies have shown consistent results in suggesting that nGVS is capable to directly alter the firing in the vestibular nerve projections and ensuing thalamocortical connections [26,41].

Noise has been commonly viewed as a hindrance to signal detection [46]. Nevertheless, recent studies suggest that the presence of a moderate amount of noise can enhance information content of a signal (e.g. trains of action potentials or neuronal assemblies signals) in non-linear systems such as sensory information processing and cortical dynamics [47,48]. Such an effect is known as the stochastic resonance (SR) phenomenon. SR has been viewed as compatible with neural models and theories of brain function [48]. Moreover, stochastic facilitation in the nervous system has shown to elicit functional benefits in sensorimotor processing [49,50]. In SR paradigm, subthreshold signals (e.g. information in the vestibular system) are encoded by threshold crossings sequence [48]. Adding noise to the subthreshold signal increases the probability of threshold crossing near the peaks of the signals, altering the sequence of threshold crossings (‘spike train’) and thus enhances the information content of sensory neural discharges [48]. A previous study found that white noise GVS may improve the locomotor performance during a short-term adaptation to visuomotor and sensory discordance [46], while another study found that pre-adaptation to two hours of pseudorandom nGVS is associated with enhanced sensorimotor performance in healthy subjects [51]. In individuals with PD, pink noise GVS was observed to decrease the amount of higher frequency components in a manual tracking task [40]. The noisy signal can be of various forms, however, a previous study has reported that pink noise is more beneficial than white noise to elicit a more beneficial neuronal information transfer in the brain [52]. Another previous study has shown that nGVS lowers the human vestibulospinal reflex threshold, resulting in the detection and transmission of input stimuli that occur below the natural vestibular threshold level which subsequently enhances the vestibular processing [53]. This enhancement is thought to be one demonstration of SR in the human vestibular system [53]. Since the vestibular system is pervasive, highly multimodal, and deeply interconnected with other areas of the brain, the enhanced vestibular processing and information transfer throughout the nervous system may possibly reflect on sensorimotor performance, in agreement with observed nGVS effects in previous studies.

Previous work employing a manual tracking task found that nGVS enhanced the speed of tracking behavior and overall damping ratio in healthy and PD subjects in certain task conditions with altered visual feedback [54]. The improvements in the tracking behavior due to nGVS were speculated to be evoked by a combination of enhanced cingulate activity due to the modulation of frontal midline theta rhythms from the vestibular stimulation, as well as an improved signal processing in the neuromotor system due to stochastic facilitation [40]. An increase of frontal midline theta activity localized at the anterior cingulate regions has been shown to correlate with a higher mental workload, decreased accuracy, and slower responses [55]. Vestibular activations have been consistently associated with the cingulate cortex in previous studies [28,32,56,57]. Further, a meta-analysis of vestibular activations has shown evidence of the activations of the caudal part of the anterior cingulate cortex (ACC), where this region is involved in motor control and attention [45]. ACC activity has been suggested to be involved in non-routine behaviors that include learning and problem-solving [58–62] and this area is associated with attention to action and response selection to new situations [60–64]. ACC is believed to contribute to monitoring tendencies of different responses in situations where errors are likely to occur [62,65]. Based on these past findings, nGVS may be able to influence fine motor control in goal-oriented tasks that require error minimization such as visuomotor tasks.

Various GVS threshold determination methods have been used in the past such as through perceived motion [36,38], cutaneous sensation [39,40], or observed body sway [66,67], with different protocols such as from lowering down from fixed suprathreshold until sensation is no longer perceived [68] or starting from imperceptible current until sensation is felt [40–42,69]. Several studies have used thresholding method through cutaneous sensation by gradually increasing current from imperceptible level until the subject feels tingling sensation under the electrodes, and observed improvement in motor and cognitive functions [40,42,54,69]. The type of stimulation used also differs based on the study objectives and are still ongoingly studied to further investigate its effects. Low amplitude currents have been used to stimulate sensory nerves and when given at suprathreshold levels above their sensory threshold, excitation occurs at the efferent terminal axon branches and myelinated afferents [70]. Several past works have used different types of stimulation and observed a range of effects in their specified behavioral observations. For examples, GVS delivery as suprathreshold sum of periodic signals improved balance function in healthy subjects [51,71], fixed (1.5 mA) DC delivery reduced subjective vertical deviation associated with spatial neglect [72], suprathreshold DC alleviated upper and lower extremities motor symptoms in PD [69], while subthreshold DC improved target detection in stroke patients [68], and subthreshold nGVS improved visual memory recall [42] and manual tracking behavior [40]. Regardless, numerous studies have shown that the signaling capacity of somatosensory afferents may be enhanced with the addition of noise stimuli delivered just at or below the perceptual thresholds [73–75].

The goal of this study is to investigate the functional effect of nGVS on fine visuomotor tracking behavior in healthy subjects. This study assessed the participants’ tracking performance during a visuomotor task to give an insight into motor coordination and accuracy [76] and broadens the findings of previous research which investigated the effect of nGVS on the manual tracking behavior of individuals with PD [40]. Participants performed a computer-based visuomotor tracking task using a joystick to follow an amplitude-modulated signal trajectory displayed on a monitor screen while simultaneously receiving either a sham stimulus or nGVS. Each participant’s noisy stimuli were generated based on their individual cutaneous threshold. Ten healthy participants completed the study performing eight trials of a 95-second tracking task, from which the trials were performed with either a sham stimulus or nGVS. Results showed that nGVS may improve the visuomotor tracking behavior with reduced error, time delay, and improved signal-to-noise ratio.

## Methods

The protocol of this study was approved by the Office of Research Ethics at Simon Fraser University and all participants signed an informed consent form.

### Participants

Ten healthy subjects (four females, six males, mean age 25.6 ± 3.5 years, all right-handed) participated in the study. Table 1 lists their demographic data.

**Table 1:** Demographic data of the participants.

Participants	Gender	Age (years)	Threshold (mA)
A01	F	25	0.04
A02	M	31	0.04
A03	F	24	0.50
A04	F	23	0.38
A05	M	28	0.14
A06	M	32	0.06
A07	F	23	0.02
A08	M	24	0.26
A09	M	22	0.14
A10	M	24	0.12

### GVS stimulus

For each participant, 16 cm^2^ pre-gelled carbon electrode pads were placed behind the mastoid area on both sides for bilateral bipolar GVS delivery. Skin surface at the electrode sites was first cleaned with a 70% alcohol prep pad to improve skin-electrode contact and avoid skin irritation. A constant current Linear Isolated Stimulator (A395R, World Precision Instruments Inc., FL, USA) was used to provide the electrical current required for stimulating the vestibular system. Each participant’s cutaneous sensory threshold was determined at the beginning of each testing session by slowly increasing the current intensity [40]. Setting the base current level at zero, direct current was applied with a stepwise 20μA increase every 20 s periods until the participant reported a tingling sensation at the electrode sites [42,77]. The current was then gradually decreased until the tingling sensation was no longer perceived. The current was then gradually increased in the same manner until the tingling sensation was felt again and this second confirmed value was used as the determined threshold of the participant. Participants were blind to the level of current stimulus throughout this procedure. The GVS threshold level of each participant is reported in Table 1 (mean 0.17±0.16 mA).

nGVS signal was generated for each participant individually using MATLAB to have a zero-mean, linearly detrended 1/f-type power spectrum [39,52] with a Gaussian distribution within 0.1–10 Hz range, and a standard deviation set to 90% of each participant’s threshold value [40]. The digital signals were converted to analog signals via NI USB-6001 (National Instruments, TX, USA) and delivered at a rate of 60 Hz to the constant current stimulator connected to the electrodes [40]. The electrodes were placed on the participants’ skin at the beginning of the session and remained there throughout the entire session regardless of the type of the trial (zero current sham stimulus or with nGVS). The participants were not informed about the order/type of trials and were told that they might feel or not feel the current stimuli at random times during the test.

### Visuomotor task

**Fig 1:**
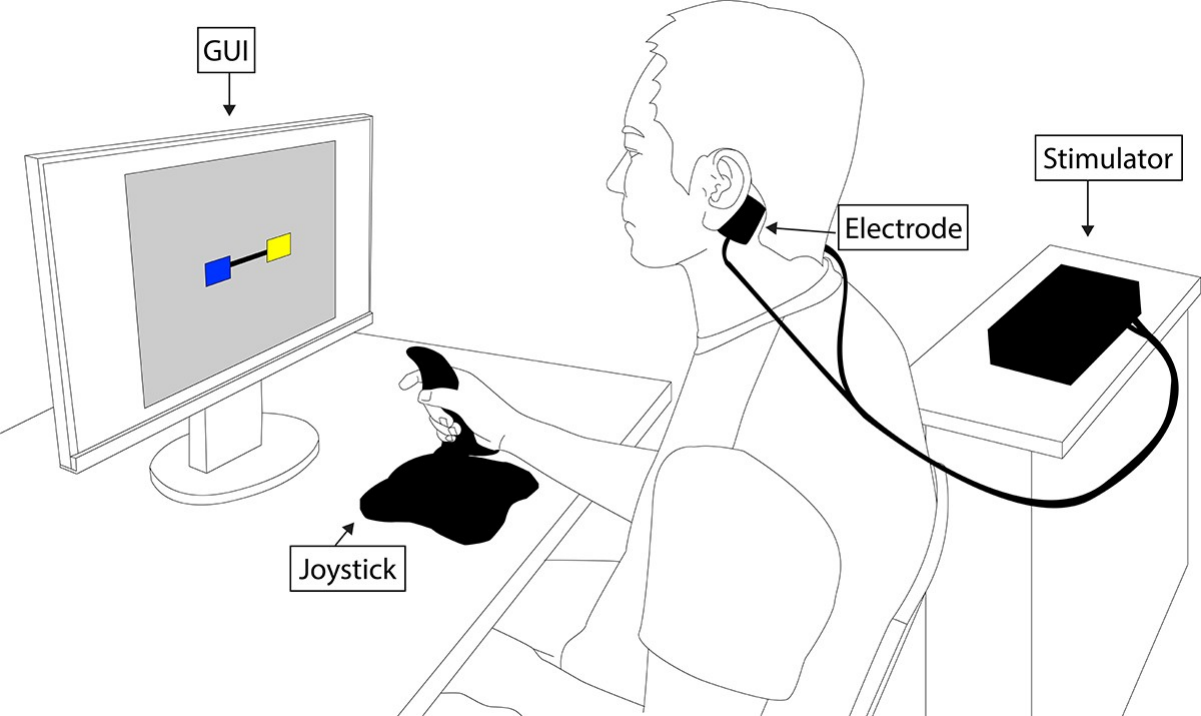
Experiment setup. Participant performing a computer-based visuomotor tracking task using a joystick with two electrode pads placed on both mastoid area for GVS delivery.

All participants performed a computer-based visuomotor tracking task while seated in front of a 27” monitor showing a graphical user interface (GUI) as shown in Fig 1. They were seated approximately 60 cm in front of the screen. The tracking task involved fine motor movements mainly generated from the wrist to control an object on the monitor using a joystick (Extreme 3D Pro Joystick, Logitech, Switzerland). The interface and experimental protocol were partially motivated by those used in another study which found that GVS influenced motor performance on manual tracking behavior in individuals with PD [40]. During the test, the screen showed a blue and a yellow box connected with a black bar. When the trial started, the blue box moved up and down to follow a pre-defined trajectory, but the movement of the yellow box was controlled by the user. The objective was to control the position of the yellow box such that the bar connecting the two boxes remains horizontal, *i*.*e*., the user-generated trajectory of the yellow box should match the pre-defined trajectory of the blue box as closely as possible. Participants used a joystick to control the vertical movement of the yellow box. The vertical tilt position of the joystick determined the speed of the controlled (yellow) box. The target (blue) and controlled boxes only move in the vertical direction. The monitor resolution was set to 1920×1080 pixels and the GUI window was sized to 1280×960 pixels. The position of the controlled box was displayed without any amplification or de-amplification on the displayed error. The speed of the controlled box was configured to be proportional to the vertical tilt position of the joystick, and since the task involved fine visuomotor tracking activity, the controlled box’s speed was limited to a maximum 8 px per sample.

**Fig 2:**
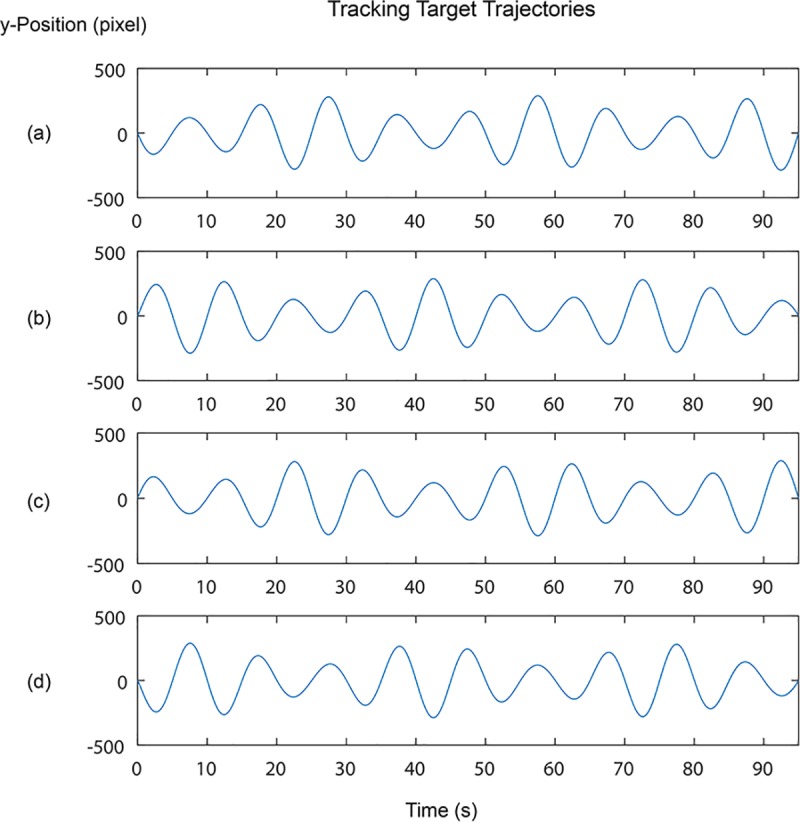
Trajectories of visuomotor tracking task. Target position to follow: (a) Trajectory 1 (b) Trajectory 2 (c) Trajectory 3 (d) Trajectory 4.

Four different waves were used as the target trajectories as shown in Fig 2. These were produced by generating an amplitude modulated signal with a carrier frequency of 0.01 Hz, a modulation frequency of 0.03 Hz, and a modulation index of 0.4/0.95. Trajectory 2 and 4 were phase shifted from trajectory 1 and 3 while trajectory 3 and 4 were inverted from trajectory 1 and 2, respectively. The slightly different trajectories were used to introduce minor variation so that the participants did not expect the same trajectories in all the trials while also keeping a consistent level of difficulty between trials. Before the test, the participant was given one practice trial to be familiar with the setup by trying out trajectory 4. The trajectories were scaled to have a peak-to-peak magnitude of 576 pixels and they were displayed at a rate of 30 frames per second. User’s trajectory was captured at the same rate as the target trajectory at 30 Hz.

### Study protocol

The cutaneous sensory-threshold value of the participant was first obtained to generate the pink noise stimulus signal specific for each participant. Current stimuli were set to zero during the sham and break periods. The pink noise stimulus during the trials with nGVS was given right at the start of the tracking task. Data were collected from each participant performing eight trials of 95 seconds visuomotor tasks with either a sham or nGVS stimulation. A rest period (no stimulation) of at least 30 seconds was given in between trials to preclude the hysteretic effects of the previous nGVS to carry over to the next trial [40]. Table 2 lists the order of the trials with the stimulation type and the trajectory used. The order of stimulation given was balanced out in such a way that for the same trajectory, two trials started without nGVS and the other two started with nGVS first. Participants were not able to see the state of the stimulator.

**Table 2:** Order of tasks with the type of stimulation and trajectory.

Trial 1	Trajectory 1	w/o nGVS
Trial 2		with nGVS
Trial 3	Trajectory 2	with nGVS
Trial 4		w/o nGVS
Trial 5	Trajectory 3	w/o nGVS
Trial 6		with nGVS
Trial 7	Trajectory 4	with nGVS
Trial 8		w/o nGVS

### Data analysis

Users performed a 95-second task for each trial. To preclude the initial tracking behavior adaptation when the task started, the first 5 seconds of the collected data were discarded, and the remaining 90 seconds of the signals were analyzed. The root-mean-square error (RMSE), time delay, and signal to noise ratio (SNR) were calculated by comparing the user’s trajectory to the target signals. The error was defined as the difference between the user’s trajectory and the target signals at each sample. We computed the time delay as the time shift that minimizes the error and was obtained by shifting the user’s trajectory signal per sample over +1 and -1 second and comparing the total absolute error. The SNR in dB was obtained by calculating the ratio of the summed squared magnitude of the target signal to the summed squared magnitude of the error. The three parameters were calculated as the following:

$$RMSE=\sqrt{\frac{\sum_{t=1}^{T}{\left(u(t)-y(t)\right)}^{2}}{T}}$$ (1)

$$Time\;Delay={\mathrm{argmin}}_{d}\sum \left|\left(u(t)-y(t-d)\right)\right|,\;d=-Fs,\ldots ,Fs$$ (2)

$$SNR=10\;{log}_{10}\left(\frac{\sum u{\left(t\right)}^{2}}{\sum {\left(u(t)-y(t)\right)}^{2}}\right)$$ (3)

where *u(t)* is the target (input) trajectory, *y(t)* is user’s (output) trajectory, *T* is the length of the signal, *Fs* is the sampling rate, and *d* is the amount of time-shift applied to the user’s signal.

The difference between without nGVS and with nGVS conditions for each trajectory was first calculated resulting in four pairwise differences between the two conditions, which were then averaged resulting in a single measure for each parameter for each participant. The outcome measures were the percentage of improvements calculated for each parameter, *i*.*e*., the ratio of the difference between with and no nGVS to the baseline of no nGVS condition. For RMSE and time delay, the difference was calculated by subtracting the with nGVS from the no nGVS condition; while for SNR, the difference was calculated by subtracting the no nGVS from with the nGVS condition. Therefore, positive percentage values represent improved performance with decreased RMSE, decreased time delay, and higher SNR in the with nGVS condition.

The entire 90 seconds duration of the signal was analyzed to evaluate the user’s overall performance. In addition, the three 30-second sub-sections of the signals were also analyzed to observe the effect of ongoing nGVS stimulation over different periods of the task.

### Statistical analysis

Statistical analysis was performed using IBM SPSS Statistics 24 on the three outcome measures, *i*.*e*., improvement percentage in RMSE, time delay, and SNR in the with-nGVS condition, as defined in the previous section. In this study, we focused on RMSE as the primary outcome measure while the time delay and SNR are the secondary measures of our findings. All statistical tests were carried out using a 95% confidence interval. Two-tailed one sample t-test against a test value of zero was employed to observe any significant difference of tracking parameters due to the with-nGVS condition. For the one sample t-test performed, the assumption of the dependent variables having a normal distribution was checked using Shapiro-Wilk’s test, while the assumption of the dependent variables not having a significant outlier was checked using Grubb’s test. Cohen’s *d* effect size was also calculated when using the one sample t-test. However, if the assumptions were not met, the two-tailed Wilcoxon’s sign rank or sign tests were utilized instead. If the data were normally distributed but had at least one significant outlier, the Wilcoxon’s sign-rank test was used. If the data were very skewed and not showing a normal distribution, the sign test was used. Significance was considered using a *p*-value of 0.05 for all the tests. Since significant differences were found on the measures using the whole 90 seconds of data, an exploratory investigation was carried out on the three 30-second subsections. Statistical tests for these subsections were performed the same way as the main outcomes.

## Results

**Fig 3:**
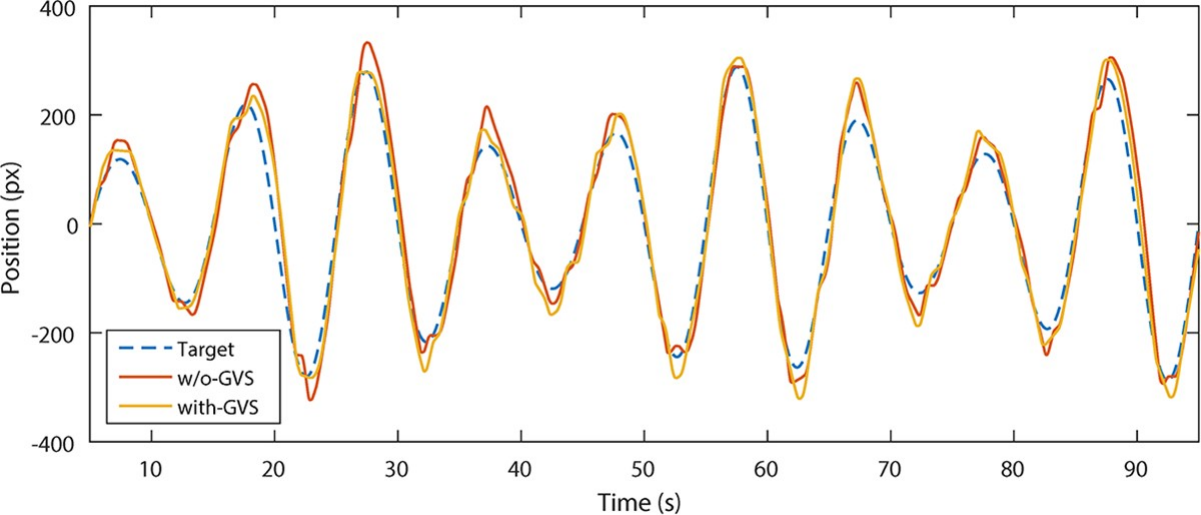
Example of collected signals (user-generated trajectory) in without-GVS and with-GVS condition while tracking a similar target trajectory.

A sample of the signals collected during one trial of with-nGVS and one trial of without-nGVS condition is shown in Fig 3. The demonstrated 90-second duration of the signal was analyzed to calculate the RMSE, time delay, and SNR.

**Fig 4:**
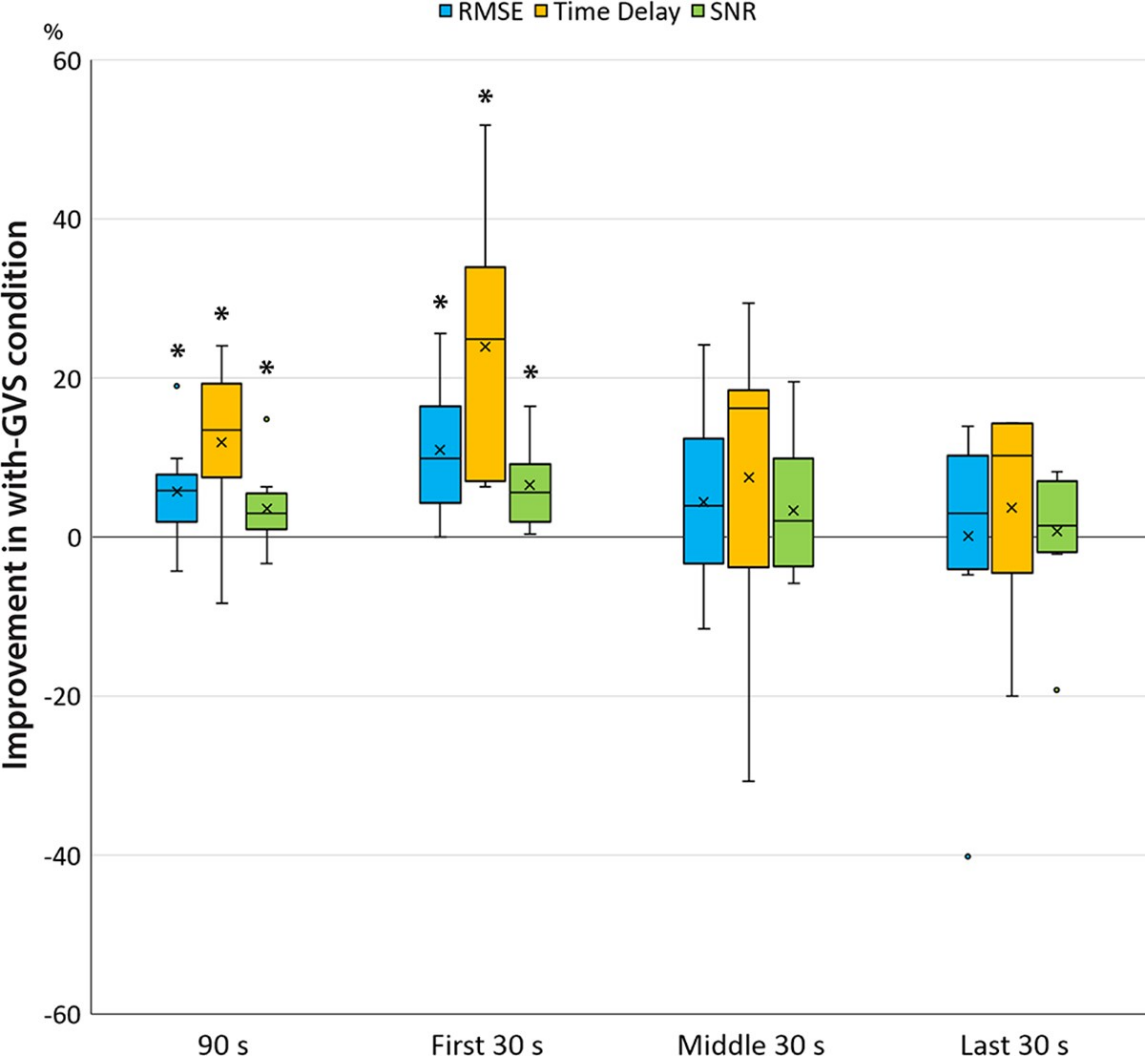
Distribution of improvement percentage in RMSE, time delay, and SNR over 90 seconds of the visuomotor tracking task and the 30-second subsections of beginning, middle, and end.

Table 3 summarizes the results of the improvement percentage in RMSE, time delay, and SNR of each subject for the entire 90-second duration and each 30-second subsection. Overall, all parameters show positive improvement with nGVS compared to without nGVS condition. For the entire 90-second signal, there are 5.71±6.20%, 11.88±9.66%, 3.57±4.78% mean improvement in RMSE, time delay, and SNR respectively. The RMSE and time delay parameters in the 90 seconds of the data showed normal distribution and no significant outlier, therefore one sample t-tests were employed. Results show significant positive improvement in decreasing the RMSE and the time delay with nGVS with a mean of 5.71±6.20% *p* = .017 and 11.88±9.66% *p* = .004 respectively. SNR measures had a normal distribution but one significant outlier, therefore, the Wilcoxon’s sign rank test was employed, and results showed a significant improvement with a median difference of 2.93%, interquartile range 3.31%, *p* = .028. Since applying nGVS significantly improved the considered outcome measures over the entire 90 s of data, further analysis was carried out by dividing the recorded duration, i.e. 90 s into three non-overlapping subsections of the 30-s signal. The rationale of this was to observe if the difference is mainly manifested at a specific period of time during the task. A significant difference was only found at the first of the three subsections, *i*.*e*., at the first 30 seconds of the signal. The RMSE and time delay showed normal distribution without any significant outlier, therefore, one sample t-test was employed. Results show significant positive improvement with decreased RMSE and time delay with nGVS with a mean of 10.99±8.76% *p* = .003 and 23.94±14.77% *p* = .001 respectively. Wilcoxon’s sign test was employed for the SNR measure, and results show a significant improvement with median 5.56%, interquartile range 3.74%, *p* = .002. No significant difference was found in the middle and last 30 seconds of the data for any of the outcome measures. Additional post-hoc analysis performed using the Holm-Bonferroni method (n = 3 for the primary parameters of 90 s data, n = 9 for the exploratory subsections data) did not affect the conclusion of the statistical tests. The results of the statistical tests in which significant differences found shown in Table 3 were marked in bold. We reported unadjusted *p*-values since the number of comparisons were relatively low and conclusions were the same, with or without the post-hoc analysis. Results show a general trend of decreased mean improvement overtime where the largest improvement was observed during the first 30 seconds while the last 30 seconds seem to show the minimal difference with GVS as shown in Fig 4.

**Table 3:** Percent improvement in RMSE, time delay, and SNR for each participant on the entire 90 seconds tracking signal and the subsections of the first, middle, and last 30 seconds.

	90 s			First 30 s			Middle 30 s			Last 30 s		
Subject	RMSE %	Time Delay %	SNR %	RMSE %	Time Delay %	SNR %	RMSE %	Time Delay %	SNR %	RMSE %	Time Delay %	SNR %
A01	7.12	12.50	3.54	10.28	28.57	4.64	5.80	17.65	2.79	5.66	0.00	2.95
A02	9.82	15.79	5.19	0.01	6.25	0.60	15.32	15.79	8.69	13.89	14.29	8.23
A03	6.28	14.29	6.29	8.98	28.95	6.42	11.41	16.67	13.40	-0.96	-18.18	-0.03
A04	5.25	11.54	2.88	10.44	20.00	6.41	-9.29	4.35	-5.61	11.86	12.00	8.13
A05	4.83	10.00	2.12	0.84	33.33	0.32	4.28	18.18	2.04	8.94	8.33	3.95
A06	19.02	19.05	14.79	25.45	6.67	16.35	24.18	19.23	19.52	9.69	0.00	6.60
A07	-4.33	24.00	-3.39	25.62	51.72	16.41	-11.60	0.00	-5.80	-40.18	12.00	-19.21
A08	-0.38	-8.33	0.03	5.41	35.71	2.29	-1.33	-30.77	-0.54	-4.76	14.29	-2.21
A09	6.82	0.00	2.97	13.36	21.05	6.69	1.53	-15.38	-3.16	0.19	-20.00	-0.14
A10	2.69	20.00	1.25	9.49	7.14	4.70	3.59	29.41	1.98	-3.86	14.29	-1.82
	**5.71**	**11.88**	**2.93**	**10.99**	**23.94**	**5.56**	4.39	7.51	3.33	2.92	10.17	1.46
	**(6.20)**	**(9.66)**	**[1.47, 4.78]**	**(8.76)**	**(14.77)**	**[2.88, 6.62]**	(10.79)	(18.38)	(8.28)	[-3.14, 9.5]	[0, 13.7]	[-1.4, 5.94]
	***t*(9) = 2.91**	***t*(9) = 3.89**	***Z***_***1***_ **= 2.19**	***t*(9) = 3.97**	***t*(9) = 5.13**	***Z***_***2***_ **=** **-2.85**						
	*p* = .017*	*p* = .004**	*p* = .028*	*p* = .003**	*p* = .001**	*p* = .002**						
	***d* = 0.92**	***d* =** **1.23**		***d* =** **1.25**	***d* =** **1.62**							

Mean (SD). Median [1^st^, 3^rd^ Quartiles]. Significant results are bolded (* *p*<0.05, ** *p*<0.01).

*p* = *p*-value, *t*(degrees of freedom) = t value, *d* = Cohen’s effect size.

*Z*_*1*_ = Wilcoxon’s sign-rank standardized test statistic, *Z*_*2*_ = Wilcoxon’s sign standardized test statistic.

## Discussions

Overall, across all subjects, a significant improvement in tracking performance with nGVS was observed during the entire 90 seconds task. Out of the ten participants, eight showed decreased RMSE, one showed larger RMSE, and one showed a minor difference in the RMSE during the with-nGVS condition. The results of this study are consistent with the SR phenomenon in which the presence of noise to sensory input can improve sensory information processing [48,78] and motor functions [46,79]. nGVS has been suggested to be able to increase neuromotor system synchronization by modulating the detection and transmission of information in the sensorimotor system through stochastic facilitation [40]. Enhanced sensorimotor performance via SR is thought to result from better sensorimotor integration and an increased cortico-muscular synchronization through the addition of noise into the sensory system [50]. Neural synchronization is a mechanism in which functionally different brain regions establish transient networks, and is believed to play an important role in cognition, perception, and action [80,81]. Since the noise levels in the brain fluctuates depending on the activity, adding moderate amounts of noise, therefore contributing to SR, could optimize task-relevant brain network formation [81]. Past studies have used various forms of noise to the sensory systems such as tactile, acoustic, visual, and electrical stimuli and suggested that SR phenomena may explain the observed enhancement of sensory and motor systems [46,50,53,81,82]. Although the SR phenomenon is ubiquitous and compatible with neural models and brain function theories [48], the exact mechanism of possible improvement in information transfer through the addition of nGVS is still unclear. Weak electrical fields from a low-intensity electrical stimulation have been shown to induce small but coherent changes in the firing rate and timing of neuronal populations, which can be magnified by a dynamic network activity [83]. However, to the best of the authors’ knowledge, the hypothesis that low amplitude nGVS modulates the spike timing to improve signal coding in the sensorimotor system has still to be fully validated in animal studies. Although the results of this work may agree with the SR phenomenon in sensory information processing [48], further studies are required to investigate in-depth the effect of nGVS at the neuronal level.

nGVS delivered at subthreshold current intensities have been previously demonstrated to be able to modulate EEG synchronization in healthy subjects, and immediately after the stimulation, nGVS predominantly leads to a mild suppression of gamma power in the lateral regions of the brain [41]. Gamma activity (31–50 Hz) has been hypothesized to play a role in sensorimotor integration or attention [84,85]. In human electrocorticography recordings, an increase of gamma activity around 40 Hz was observed during the performance of visuomotor tasks such as threading pieces of tubing or moving a cursor to a visual target [86]. In another study, a higher peak of 40 Hz event-related synchronization (ERS) was observed during a task in a higher memory-load condition [87]. There is a possibility that the observed improvement in this study was influenced by the nGVS that may have suppressed the increased gamma activity induced by the visuomotor task. Further work is required to investigate this hypothesis to gain a better understanding of the causal effect of nGVS and gamma activity related to cognitive load induced during the performance of a visuomotor task.

nGVS has been shown to directly alter the firing in the vestibular nerve projections and ensuing thalamocortical connections [26,41]. A previous study has identified a thalamocortical tract between the anterior thalamic nuclei and cingulate gyrus in the human brain [88]. The ACC is associated with mental workload, response time, accuracy, motor control, and attention [40,45,55]. With regard to this, cortical activity is also dynamically modulated from the basal forebrain and the brainstem by neurotransmitter-specific pathways such as the cholinergic and dopaminergic pathways which innervate the ACC and project back to the basal forebrain [89–94]. The basal forebrain cholinergic system acts to improve sensory coding during aroused and attentive states resulting in an enhanced sensory perception [95]. The dopaminergic pathways which innervate the basal ganglia-thalamocortical circuit model, are involved in goal-directed behavior, executive motor functions and planning, and learning processes [96]. In a goal pursuit task, ACC has been shown to provide a top-down modulating signal to the ventral tegmental area (VTA), a region that transmits a major source of dopamine to the basal ganglia through the mesolimbic pathway where this pathway is associated with motivation and reward-related motor learning [97–101]. The observed improvement in the motor behavior in this study may also be based on the modulation of ACC activity from the nGVS, influencing the cholinergic and dopaminergic system and subsequently affecting the sensory perception and motor function in this goal-oriented visuomotor task. Although overall improvement in tracking behavior with nGVS was observed in this study, a future investigation is required to test this hypothesis to further understand the effects of nGVS on the dopaminergic pathways since excessive excitatory activity in the pathways may result in unwanted motor action.

The differences in the RMSE improvement were about two times larger (10.99±8.76% vs 5.71±6.20%) during the first 30 seconds compared to the entire task. On the other hand, the second subsection showed relatively less improvement while the last subsection showed the least mean difference in the RMSE improvement. This finding may suggest that the visuomotor tracking behavior improvement due to simultaneous nGVS is mainly observed over the initial adaptation period when the participants start to receive the stimuli during the beginning of each trial. There is still little known regarding the effect of duration on the immediate implication of nGVS delivery. A previous study employing 10-min white noise transcranial random current stimulation (tRNS) over the motor cortex observed consistent cortical excitability increases lasting 60 min after stimulation [102], while another study employing 4-min pink noise tRNS over the left-hemispheric sensorimotor area observed a transient reduction in blood oxygenation level dependent (BOLD) response in the sensorimotor cortex [103]. Past results indicate that tRNS applied with different durations in combination with a task may result in different outcomes [103].

Norman-Shallice proposed a model of executive control framework that could provide a possible explanation to the observed decreasing improvement over time. The framework is governed by a contention-scheduling which corresponds to routine selection, and a supervisory attentional system that is necessary for successful task completion during an inadequate routine process such as decision making, error correction, novel responses, conditions perceived difficult, and overcoming habitual responses [104]. Past studies have observed an increased ACC activity during planned voluntary arm movements compared to that during resting, learned, fixed sequence of movements, or in highly-learned situations [105–107]. According to this model, the activity of ACC is associated with an active supervisory system, and it diminishes or disappears as the tasks become routine [63]. The cingulate is believed to play an important role for voluntary reactivation of the brain areas that are automatically activated by visual input in an error-correction task [63]. The decreasing trend of improved performance in this study could suggest that GVS may have a more pronounced effect during a situation requiring more novel responses and that its effect diminishes gradually as the task is perceived to be more routine.

On the other hand, physiological studies have shown that cortical response adapts to stimuli such as visual [108] and somatosensory stimuli [109,110]. The sensory systems adapt their sensitivity to the ambient stimulation to improve their responsiveness to changes in stimulation [111]. For example, tactile fibers become desensitized when the skin is subjected to prolonged vibrotactile stimulation over an exponential time course with a time constant of about 10 s [112]. The time course of electrically induced sensory adaptation, ranging from 10 to 100 s, has been found to be very similar to mechanically induced counterparts [113]. Both electrically and mechanically induced adaptations are believed to not be governed merely by the mechano-transduction process, but rather by a downstream mechanism such as the increase in spike generation threshold in the central nervous system [113–115]. During rapid changes in sensory input, synaptic depression of thalamic input to the cortex has been shown to contribute to the dynamic regulation of neuronal sensitivity [116], and that nGVS is thought to influence the thalamic processing through the manipulated thalamocortical loops receiving input from the vestibular afferent projections [41,44,45]. There could also be a possible unknown neural adaptation mechanism to SR phenomenon in the nervous system due to a given duration of nGVS delivery that may contribute to the diminishing trend of improvement over time during the task. More comprehensive studies are required to better characterize the effect of nGVS duration in a similar task behavior.

The different trajectories were used to introduce minor variation and perception of a randomized target so that the participants would not expect the same target trajectory and the learning effect was minimized throughout the study. However, the characteristics of the trajectories in terms of their frequency and magnitude were identical so that the level of difficulty was consistent between all the trials. The only differences were the starting point of the trajectory (shifted) and an inverted signal for different starting slope, although some participants found some trials to be more challenging albeit the minor differences between the trajectories. The four target trajectories in this study were used twice each for the without and with-GVS condition, and the average over the differences between the two conditions was taken as the outcome measures to represent participant’s overall performance, based on the rationale to eliminate the variable of tracking difficulty in comparing the two conditions. Since the averaged measures were used in which two trials started without-GVS and the other two started with-GVS, learning effects did not manifest in the outcome measures used in the statistical analyses.

Individual’s threshold for electrical stimulation is determined empirically by clinicians, practitioners, or researchers and is inherently subjective based on their perceived motion/tingling sensation or researcher’s qualitative assessment such as through observed body sway. Consequently, this could be one of the many factors that are partly conducive to the variability in the overall observed effect of GVS. In this study, threshold level determination relied on participant’s reported feedback of the perceived cutaneous sensation like tingling or itching. Two participants’ reported threshold values (0.38, 0.50 mA) were higher than the mean±SD (0.17±0.16 mA) of all the ten participants, although none of them was a significant outlier based on Grubb’s outlier test. Cutaneous threshold level from electrical stimulation varies between individuals due to many factors such as different proportion of muscle to adipose mass [117], skinfold thickness, muscle cross-sectional area [70], and myelinated fiber diameters [118,119] which affect how fast sensory information travels to the brain and spinal cord [120]. The myelin-insulated nerve fibers Aβ and Aδ are responsible for carrying somatosensory and nociceptive information [121,122] and mediating the sensory threshold perception commonly reported as tingling or itching sensation [123]. The activation of Aβ afferents [118,119] and somatic sensory receptors at cutaneous and subcutaneous tissues [14,70] vary between individuals due to their underlying physiological differences which contribute to the inter-subject threshold variability. In addition to the estimated threshold variation, skin and bones conductivities, as well as internal body resistance are among other factors that affect how the electrical current travels through the body [124] which consequently influence the penetration of the external stimulation to each individual’s vestibular system.

Several previous works found improvement in motor and cognitive functions due to potential SR phenomenon using noisy stimulus with the magnitude to be at or below the subject threshold [36,39,42,66]. This study was designed to investigate the effect of pink noise GVS generated based on subject’s cutaneous threshold that was obtained through gradual increase of stepwise current and relied on subject’s feedback on perceived tingling sensation under the electrodes. The generated pink noise signal used in this study was based on 90% of the subject’s cutaneous threshold similar to the previous study that observed improvement in manual tracking behavior in PD subjects [40]. However, the optimal stimulus could also possibly depend on the type of stimulus, the thresholding method, and the type of task to be performed. For example, a white noise 0–30 Hz stimulus nearly half of perceptual threshold that was determined using periodic signal until subjects perceived motion was suggested as an optimal stimulus for overall improvement in locomotor stability and balance function [36,38]. However, another study employed pink noise 0.01–2 Hz stimulus at 60% of the nociceptive threshold and found improvement in motor functions in individuals with neurodegenerative disease [125]. Regardless, the various types of thresholding protocols and stimuli used in the past such as by using sinusoidal signals, white noise, pink noise, and DC current contribute to a better understanding of effects of different types of GVS stimuli. This study was limited to only using one specific stimulus for each subject and further work requires an investigation of the optimal stimulus related to a specified thresholding protocol to be used in a similar visuomotor task. Additionally, GVS has been shown to induce a wide range of effects including postural, ocular, and perceptual responses on certain current intensities [24,26]. For example, GVS applied during standing with eyes closed at 0.7 mA current intensity has been shown to induce a tilt of head relative to the torso at less than about 0.5 degrees, and a significant decrease in tilt (*p*<0.01) occurred when subjects were seated [126]. Another study reported ocular torsion and perceptual tilts at preferred current intensities of 1–3 mA in which slight static ocular torsion (1.0±0.4 and 1.2±0.3 degree) was observed using 1.0 mA left and right anodal GVS respectively and no nystagmus was seen with currents of less than 3 mA [127]. However, the nGVS used in this study was delivered at relatively low intensities with a mean±SD of 0.17±0.16 mA. This current is presumably not sufficiently high to induce notable postural, ocular, and perceptual disturbances in performing the task.

It is also important to note although overall improvements in many aspects were observed in previous studies, not every single subject showed improvement. For example, although eight participants showed positive improvements in RMSE in the overall duration of the task, one subject showed negative improvement (-4.33%) and one subject showed a minimal difference (-0.38%). The two subjects showing negative RMSE improvement did not correspond to the two subjects with the threshold higher than the mean±SD over the ten participants, and that the R^2^ value between RMSE improvement over the whole period of the task and threshold values of participants showed to be 0.01. Non-uniform improvements have also been observed in the previous study using nGVS where three out of thirteen participants did not show improvements in locomotor stability [36] and in another study, 30% of the participants did not show improvement in their balance function [38]. In this study, the generated pink noise stimuli had zero mean, 1/f-type power spectrum with Gaussian distribution with standard deviation of 90% participant’s cutaneous threshold, yielding ~26% of the signals to have a magnitude higher than the cutaneous threshold. Although the stimuli were delivered at the rate of 60 Hz, the participants might have still perceived several of the higher peaks of the stimulus. The same study which observed improvement in locomotor stability using various magnitudes of nGVS found that two subjects showed improvement with suprathreshold current [36] while other study using GVS twice the threshold found improvements in motor symptoms in individuals with PD [69]. These results encourage further work to investigate the applicability of GVS beneficial effect in a wider population, as well as optimizing the type of subject-specific stimulus more objectively to achieve more consistent positive outcomes in the future.

Other limitations of this study include the small sample size and narrow age range group of the participants. Adding more subjects in a wider age range group will allow for a more comprehensive assessment of the validity and repeatability of the observed improvement due to the nGVS. All the participants involved in this study were right-handed, therefore further study may also investigate the effect of GVS while performing a similar task using their non-dominant hand. Since the nGVS was zero-mean and delivered in a bilateral bipolar manner, we expect the participants to show a similar trend if their dominant hand were the left hand, but this point needs further verification to be addressed in future studies. A previous study has shown that most right-handed individuals had better accuracy in directing their gaze in the left visual hemifield than the right which suggested that their right visual hemifield is more superior in the oculomotor control function (44). A simultaneous dual-tracking task using both hands may also be conducted in the future to investigate similar tracking behavior due to nGVS in both dominant and non-dominant hands.

This study mainly used RMSE as a primary measure of the outcome, which was partly related to the secondary measures of time delay and SNR since they also used the error calculated as the difference between the target and user’s position. Other outcome measures may be explored to characterize the quality of the tracking behavior between the two conditions. In this study, improvement in RMSE and SNR over the whole period of the task showed R^2^ value of 0.93 while between RMSE and time delay, R^2^ value was 0.02 since the latter used the time-domain parameter. Future works may also study the effect of nGVS delivered at various magnitudes and using different trajectory paths to follow. A double-blind sham-controlled study that employs noisy stimulation limited to the sub-cutaneous threshold may be required to further assess the validity of simultaneous nGVS effect on the visuomotor performance in a more rigorous way. One of the criteria for SR to occur is that it requires weak sensory input signal [47], future mechanistic study at a neuronal level is required to fully understand the implications of the vestibular signals throughout the sensory information processing system in a visuomotor task. The task in this study involved a fine motor movement, further investigation of the effect of nGVS on gross motor skills may also be conducted to study if similar findings could be observed. This could broaden the knowledge of using stochastic GVS as a means to improve sensorimotor performance in a wider range of applications.

In addition, the aging process is also accompanied by a general slowing of information processing and longer reaction time [128]. Several studies have shown that general motor skills and motor learning ability decline as people age [129–131], while another study showed that seniors demonstrate impairments in performing a visuomotor task with longer response time [132]. Spatial deficits such as reduced spatial attention and short-term memory, occur in conjunction with nonspatial deficits such as reduced target detection, orientation, and vigilance as observed in individuals with unilateral spatial neglect [133]. A deterioration in spatial coordination of finger and wrist movements, as an example, has also been suggested to affect fine motor performance such as during a handwriting task [134]. Since this study shows promising results of improved fine visuomotor tracking behavior in the healthy non-senior population, future works may also consider a similar study on the aging population as well as individuals with spatial deficits.

## Conclusions

This study demonstrated that nGVS might be beneficial in improving sensorimotor performance in a computer-based fine tracking task in healthy subjects. The task required participants to control an object following an amplitude-modulated signal target path displayed on a monitor by using a joystick. We found that simultaneous nGVS delivery improved tracking performance with an overall decreased mean of 5.71±6.20% in RMSE and 11.88±9.66% in time delay as well as an increased median of 2.93%, IQR 3.31% in SNR. The results of this study suggested the potential for future development in using simultaneous nGVS delivery to assist in daily activities such as driving, operating machines and other activities requiring fine movement control such as in arts, sports, and telerobotic applications. This study is focused on observing the participant’s tracking behavior and does not investigate the neuronal mechanism of the brain due to the nGVS delivery while performing the fine motor task. Since the outcome of this study is promising, a mechanistic study following this work is suggested for future research.
